# Preliminary Cold Tolerance Evaluation of Seven *Ilex* Species Based on Physiological Responses of Detached Leaves to Acute Low-Temperature Stress

**DOI:** 10.3390/plants15111751

**Published:** 2026-06-04

**Authors:** Bo Lu, Xiaolong Wang, Xinran Chong, Haoran Jia, Chuanyong Wang, Hong Chen, Ting Zhou

**Affiliations:** 1 Institute of Botany, Jiangsu Province and Chinese Academy of Sciences, Nanjing 210014, China; lubo@jib.ac.cn (B.L.);; 2 Jiangsu Key Laboratory for the Research and Utilization of Plant Resources, Nanjing 210014, China

**Keywords:** *Ilex* genus, evergreen species, cold tolerance, physiological response

## Abstract

The genus *Ilex* L., the sole member of the family *Aquifoliaceae*, is valued for its high ornamental value. However, low winter temperatures restrict the distribution of its evergreen species in colder regions. In this study, detached leaves of seven evergreen *Ilex* cultivars were subjected to acute low-temperature stress, and key physiological parameters (cell membrane permeability, osmoregulatory substances, and chloroplast pigments) were measured. The results showed that under low-temperature stress, relative electrical conductivity (REC) and malondialdehyde (MDA) content increased with decreasing temperature, while soluble protein (SP), soluble sugar, and free proline (Pro) contents first increased and then decreased. A positive association was observed between REC and MDA, as well as between REC and SP, while REC showed a negative association with Pro. Furthermore, random forest analysis indicated that MDA, proline, and chlorophyll a together accounted for 72.6% of the variance in REC. These findings demonstrate the physiological responses of detached leaves of evergreen *Ilex* species to acute low-temperature stress and offer an initial assessment of their cold tolerance.

## 1. Introduction

The genus *Ilex* L., the sole member of the family Aquifoliaceae, comprises approximately 600 species widely distributed across tropical, subtropical, and temperate regions [1]. This genus includes both deciduous and evergreen shrubs or trees. Evergreen species are characterized by year-round lush foliage and distinctive berry-like drupes in diverse colors that often persist through winter, creating striking visual contrast in deciduous landscapes and conferring high ornamental value [2]. Additionally, *Ilex* species possess significant economic value, with their wood, leaves, and fruits being extensively utilized in beverage preparation, pharmaceutical development, and wood processing [3]. Due to regional temperature variations, evergreen *Ilex* species are predominantly distributed in warm low-to-mid latitude areas. In contrast, evergreen broad-leaved plant resources are scarce in cold regions and higher latitudes, resulting in monotonous winter landscapes. *Ilex* species harbor considerable cold tolerance potential, offering possibilities for the landscape application of evergreen hollies in colder high-latitude regions [4].

Low temperature is one of the most harmful environmental stressors encountered by vascular plants, limiting their geographical distribution worldwide [5]. To adapt to low temperatures, plants have evolved complex morphological, physiological, and biochemical adaptive processes throughout their long-term evolution [6]. Understanding these adaptive processes under controlled conditions often relies on the use of detached leaves [7,8], cuttings [9], and branches [10], which have been widely employed for cold tolerance assessment in various plant species. The cell membrane is the primary site affected by low temperature [10]. According to the membrane lipid phase transition hypothesis, low temperature increases membrane permeability, leading to electrolyte leakage from cells, which is manifested as an increase in relative electrical conductivity (REC) [11]. Based on this principle, the low temperature 50% lethal (LT_50_) is an indicator commonly used to reflect plant cold tolerance thresholds and has been widely applied in cold tolerance evaluation across various plant species [7,12]. Previous studies on cold tolerance in *Ilex* species using LT_50_ have reported that female plants of *Ilex centrochinensis* exhibited stronger cold tolerance than male plants [8].

The disruption of intracellular reactive oxygen species (ROS) homeostasis is a key consequence of low-temperature stress, as excess accumulated ROS triggers membrane lipid peroxidation [13]. The content of malondialdehyde (MDA), the end product of this process, reflects the extent of membrane system damage [14]. Therefore, maintaining ROS homeostasis is crucial for plant survival under adverse conditions. To counteract oxidative damage, plants activate an enzymatic defense system centered on superoxide dismutase (SOD) and peroxidase (POD) [15]. In addition to oxidative stress responses, osmotic regulation is another key strategy for plants to adapt to low temperatures. This process involves the accumulation of osmotic regulators, including proline (Pro), soluble sugars (SS), and soluble proteins (SPs), to balance intracellular and extracellular osmotic pressure, lower the freezing point of cells, and enhance water retention capacity [16,17]. Previous studies on various woody plants have suggested that the physiological response to low-temperature stress involves a network of multiple co-regulated processes, indicating that a multi-indicator approach is beneficial in cold tolerance assessments.

Notably, existing reports have indicated that certain *Ilex* species, such as *I. verticillata, I. fargesii,* and *I. bioritsensis,* exhibit considerable cold tolerance, enabling them to overwinter successfully in open fields across some northern Chinese regions where minimum winter temperatures range from −15 °C to −25 °C. This characteristic offers a promising solution to the long-standing scarcity of evergreen foliage plants for landscaping in cold climates [1]. However, the physiological responses of evergreen *Ilex* species to low temperatures remain poorly understood, and the evaluation of cold-tolerant varieties is still insufficient. Consequently, in-depth investigations into the cold tolerance of *Ilex* species are needed to support their landscape use and ecological function in high-latitude and cold regions.

In this study, detached leaves of seven evergreen *Ilex* species were subjected to short-term low-temperature treatments under controlled conditions. Multiple key physiological parameters were measured under low-temperature treatments, including cell membrane permeability, osmoregulatory substances, and chloroplast pigments. This study aims to physiologically assess the responses of detached leaves of evergreen *Ilex* species to acute low temperatures and to preliminarily evaluate their cold tolerance.

## 2. Materials and Methods

### 2.1. Experimental Materials

Three-year-old, robust, pest-free, and uniformly growing cutting-propagated seedlings of seven evergreen Ilex cultivars were collected from the Holly Nursery of the Ornamental Plant Breeding Group, Institute of Botany, Jiangsu Province and Chinese Academy of Sciences (Nanjing, China; 32°03′ N, 118°49′ E), a region characterized by a humid subtropical climate with winter minimum temperatures typically ranging from −5 °C to −8 °C. The cultivars included: *Ilex opaca* Aiton, *Ilex* × *cassine*, *Ilex cornuta* Lindl. et Paxt. ‘Lutea-carpus’, *Ilex cornuta* ‘Dwarf Burfordii’, *Ilex* ‘Nellie R.Stevens’, *Ilex* × ‘China Girl’, *Ilex dabieshanensis*. All plants were grown outdoors in the nursery for three years under natural light conditions, exposed to natural winter conditions without any protective measures (e.g., cold frames, mulch, or greenhouse covering). Regular irrigation was applied to keep the soil moist, and compound fertilizer was applied quarterly according to standard nursery practice.

### 2.2. Experimental Design

Mature leaves (the 2nd to 5th leaves from the apex) were randomly collected from branches of the seven Ilex cultivars. The leaves were rinsed three times with deionized water, blotted dry with absorbent paper, and set aside. The cleaned leaves were wrapped in gauze moistened with deionized water to a saturated but not dripping condition, placed in resealable plastic bags that were not vacuum-sealed, allowing minimal air exchange, and pre-cooled overnight in a refrigerator at 5 °C. For each temperature treatment, three biological replicates were performed, with each replicate consisting of leaves collected from three different individual plants per cultivar.

The pre-cooled samples were then transferred to a programmable low-temperature circulator (DC-2030C, TenLin Instrument Inc., Changzhou, China) programmed to decrease the temperature linearly at 0.8 °C·min^−1^ from the control temperature of 5 °C to −20 °C, with intermediate set points at 0 °C, −5 °C, −10 °C, and −15 °C. The cooling rate was consistent across all temperature gradients. Upon reaching each target temperature, the samples were maintained for 2 h in darkness before one set of samples was removed. This process was continued until samples from all six temperature set points had been collected. To minimize the risk of ice formation, the circulating bath medium (ethylene glycol-water mixture) prevented direct ice contact with the samples. After the final treatment, all samples were thawed in a 5 °C refrigerator for 12 h. Upon thawing, leaf discs of 6 mm diameter were punched from the treated leaves using a cork borer, and all subsequent physiological measurements were performed on these discs. All sample handling and storage were conducted in the dark to avoid light interference.

### 2.3. Measurements of Physiological Parameters

REC was measured using a DJS-11D conductivity meter (Shanghai Leici Instrument Co., Ltd., Shanghai, China) following the method described by Zhou and Leul [18]. Leaf discs (6 mm in diameter) were punched from the treated leaves using a cork borer. For each treatment group, 0.2 g of leaf discs were weighed using an electronic balance, placed into test tubes with stoppers, and immersed in 10 mL of deionized water. After thorough shaking, the samples were allowed to stand for 30 min, and the initial electrical conductivity (R1) was measured. The test tubes were then boiled in a water bath for 20 min, cooled naturally to room temperature, and the final electrical conductivity (R2) was measured. REC, representing cell membrane permeability, was calculated using the following formula:

REC = R_1_/R_2_ × 100%

LT_50_ was calculated by fitting REC to the Logistic regression equation: y = k/(1 + ae^−bx^), where a, b, k > 0. In this equation, y represents the rate of cell injury, x is the treatment temperature, k is the saturation capacity of cell injury rate, and a and b are equation parameters. In this study, k was set to 100, as REC theoretically approaches 100% when cells are completely damaged [9]. To determine the values of a and b, the equation was linearized to: ln[(k − y)/y] = lna − bx. By setting y_1_ = ln[(k − y)/y], the relationship between cell injury rate (y_1_) and treatment temperature was transformed into a linear equation. The values of a and b, along with the correlation coefficient R, were obtained through linear regression. The inflection point of the curve was taken as the half-lethal temperature (LT_50_), calculated as LT_50_ = lna/b, with R^2^ representing the goodness of fit [12].

MDA content was extracted and quantified using the thiobarbituric acid method [19]. SS content was determined by the anthrone colorimetric method [20]. SP content was measured with Coomassie Brilliant Blue G-250 [21], while Pro content was analyzed via ninhydrin colorimetry [22]. Chlorophyll content, including chlorophyll a (Chl *a*), chlorophyll b (Chl *b*), and carotenoids (Car), was extracted and determined using the ethanol extraction method [23].

### 2.4. Statistical Analyses

All determinations were performed using three biological replicates. Before parametric analyses, the data were tested for normality using the Shapiro–Wilk test and for homogeneity of variances using Levene’s test. Data meeting these assumptions were subjected to one-way analysis of variance, followed by Duncan’s multiple range test at a significance level of *p* < 0.05. Statistical analyses were conducted using SPSS version 27.0 (IBM Corp., Armonk, NY, USA). Graphical representations were generated using GraphPad Prism version 9.0 (GraphPad Software, San Diego, CA, USA).

Random forest analysis was applied using Python 3.6 (Python Software Foundation, Wilmington, DE, USA) following the method described by Li et al. [24]. All indicators were normalized using min-max scaling. The dataset was divided into training (70%) and testing (30%) sets using the train_test_split function, ensuring each cultivar and temperature treatment was represented in both subsets. A 10-fold cross-validation was used to train the model. Key model parameters were set as follows: n_estimators = 100 (optimized over a range of 0–200), min_samples_split = 2, min_samples_leaf = 1, and max_features = “auto” (Table S1). Model evaluation results, which demonstrated satisfactory accuracy, are presented in Table S2.

## 3. Result

### 3.1. Response of Membrane Stability to Low Temperature

**Figure 1:**
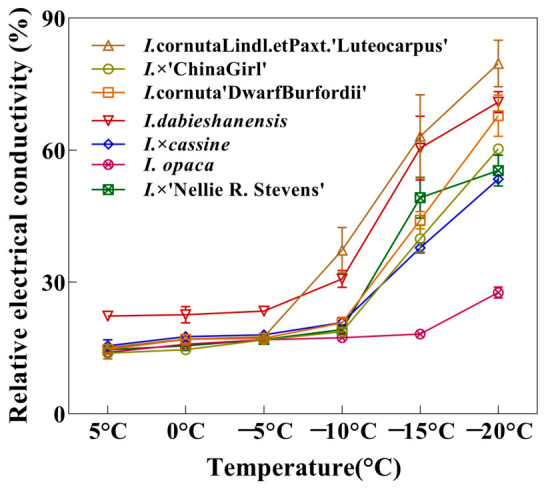
Variation in relative electrical conductivity of leaves among seven *Ilex* species under low-temperature stress. Error bars represent standard deviation (SD) of three biological replicates. The same applies to Figure 2, Figure 3 and Figure 4.

Under low-temperature treatments, the REC in the leaves of all seven *Ilex* cultivars generally increased as the temperature decreased (Figure 1). From 5 °C to −10 °C, REC showed relatively minor changes. By contrast, temperatures below −10 °C induced a sharp increase in REC for all cultivars except *I. opaca.* Specifically, at −20 °C, *I. opaca* exhibited the lowest REC (27.61%), significantly lower than that of the other cultivars.

**Figure 2:**
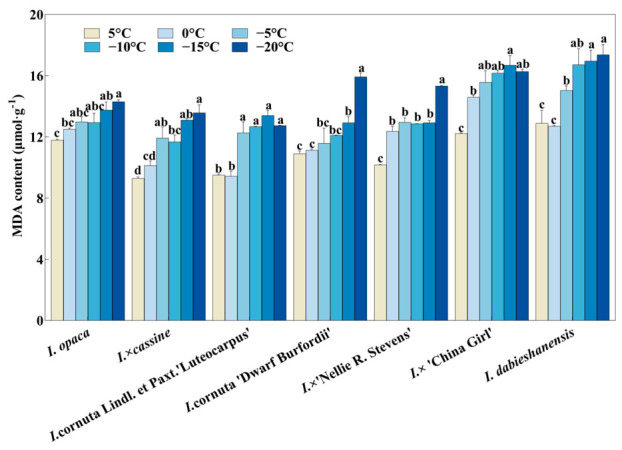
MDA content of leaves among seven *Ilex* species under low-temperature stress. Lowercase letters indicate significant differences among different temperatures within the same cultivar (*p* < 0.05). MDA, malondialdehyde.

**Figure 3:**
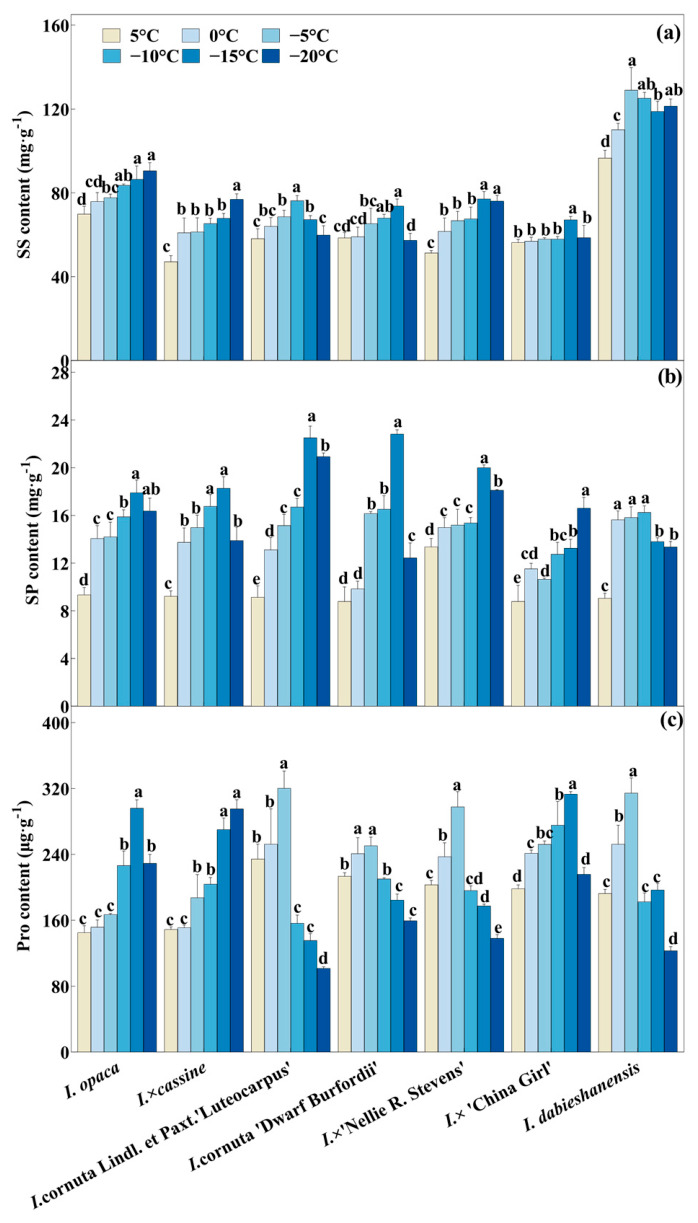
Changes in SS (**a**), SP (**b**), and Pro (**c**) contents in leaves of seven *Ilex* species under low-temperature stress. Lowercase letters indicate significant differences among different temperatures within the same cultivar (*p* < 0.05). SS, soluble sugar; SP, soluble protein; Pro, proline.

**Figure 4:**
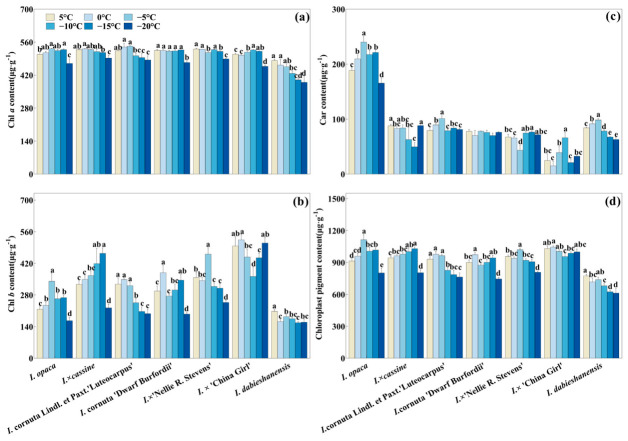
Variation in Chl *a* (**a**), Chl *b* (**b**), Car (**c**) and total chloroplast pigment content (**d**) of leaves among seven *Ilex* species under low-temperature stress. Lowercase letters indicate significant differences among different temperatures within the same cultivar (*p* < 0.05). Chl *a*, chlorophyll a; Chl *b*, chlorophyll b; Car, carotenoids.

The MDA content in the leaves of all seven *Ilex* cultivars progressively increased with decreasing temperature. *I. dabieshanensis* accumulated the highest MDA content (17.37 μmol·g^−1^) at −20 °C, with a pronounced upward trend throughout the cooling process. *I. cornuta* ‘Dwarf Burfordii’ and *I.* ‘Nellie R.Stevens’ exhibited a gradual increase in MDA from 5 °C to −15 °C, followed by a sharp rise at −20 °C. In *I. cornuta* Lindl. et Paxt. ‘Lutea-carpus’, MDA increased substantially from 0 °C to −5 °C but remained relatively stable between −5 °C and −20 °C. MDA content in *I. × cassine* and *I. opaca* increased continuously with decreasing temperature (Figure 2).

The LT_50_ values derived from REC measurements are presented in Table 1, with R^2^ values ranging from 0.77 to 0.90, indicating generally acceptable model fitting. For *I. opaca*, no inflection point was observed within the tested temperature range (5 °C to −20 °C); therefore, its LT_50_ was not derived. For the six cultivars that reached an inflection point, LT_50_ values ranged from −22.6 °C to −11.9 °C, with *I. × cassine* showing the lowest value and *I.* cornuta Lindl. et Paxt. ‘Luteacarpus’ showing the highest value.

### 3.2. Response of Osmotic Regulatory Substances to Low Temperature

For seven species, SS, SP, and Pro generally showed trends of gradually increasing or first increasing and then decreasing with decreasing temperature, yet interspecific patterns varied (Figure 3). Regarding SS content, *I. dabieshanensis* significantly increased (by 34.7%) from 5 °C to −5 °C and then decreased (by 9.4%) after −10 °C. *I. cornuta* Lindl. et Paxt. ‘Lutea-carpus’ and *I. cornuta* ‘Dwarf Burfordii’ peaked at −10 °C and −15 °C respectively, and then declined significantly. In contrast, *I. × cassine*, *I. opaca*, and *I.* ‘Nellie R.Stevens’ showed progressively higher SS levels as temperature decreased, with increases of 63.02%, 29.54%, and 48.12%, respectively, at −20 °C (Figure 3a). SP content measurements indicated that *I.* × ‘China Girl’ exhibited a continuous increase with decreasing temperature, with a substantial rise at −20 °C compared to the 5 °C baseline. The remaining varieties initially increased and then decreased: *I. dabieshanensis* peaked at −10 °C and then declined at −20 °C; the other five varieties generally reached high SP levels at −15 °C and then declined to varying degrees at −20 °C, with *I. cornuta* ‘Dwarf Burfordii’ showing the largest decrease and *I.* ‘Nellie R.Stevens’ the smallest (Figure 3b). Pro content measurements revealed that *I. × cassine* showed a continuous increase with decreasing temperature, peaking at −20 °C. The remaining varieties initially increased and then decreased: *I. opaca* and *I.* × ‘China Girl’ peaked at −15 °C and then declined at −20 °C; the other varieties peaked at −5 °C and then declined significantly at −20 °C (Figure 3c).

### 3.3. Response of Chloroplast Pigment Content to Low Temperature

Under low-temperature stress, the chlorophyll pigment contents in the leaves of the seven *Ilex* cultivars exhibited distinct variation patterns. With the exception of *I.* × ‘China Girl’, *I. dabieshanensis*, and *I. cornuta* ‘Dwarf Burfordii’, the pigment contents in the remaining four cultivars initially increased and then decreased as the temperature declined. Nearly all cultivars reached their minimum pigment levels at −20 °C. Notably, *I. dabieshanensis* maintained significantly lower chlorophyll pigment contents throughout the stress period compared to the other cultivars (Figure 4d).

For Chl *a*, most cultivars displayed a trend of either increasing first and then decreasing or gradually decreasing with declining temperature. At −20 °C, the Chl *a* content in all cultivars was significantly lower than at other temperatures (Figure 4a). Compared with Chl *a*, Chl *b* showed greater variation across different temperatures, with *I. dabieshanensis* exhibiting significantly lower Chl *b* levels than the other cultivars (Figure 4b). In contrast, the Car content varied inconsistently among cultivars with no clear trend in response to temperature change. Notably, *I. opaca* exhibited significantly higher Car content than the other cultivars (Figure 4c).

### 3.4. Relationship Between Cell Membrane Permeability, Osmoregulatory Substances, and Chloroplast Pigments and Cold Tolerance

Correlation analysis was performed on the physiological indicators of the seven *Ilex* cultivars under low-temperature stress (Table 2). The results revealed that REC was significantly (*p* < 0.01) positively correlated with MDA and SP, and significantly negatively correlated with Pro. Additionally, REC showed significant negative correlations with Chl *a* and Chl *b*. Significant correlations were also observed among other physiological indicators. Specifically, Chl *a* was significantly positively correlated with Chl *b* and significantly negatively correlated with MDA and SS. Chl *b* exhibited significant negative correlations with Car and SS, and a significant positive correlation with Pro. MDA was significantly positively correlated with SS.

**Figure 5:**
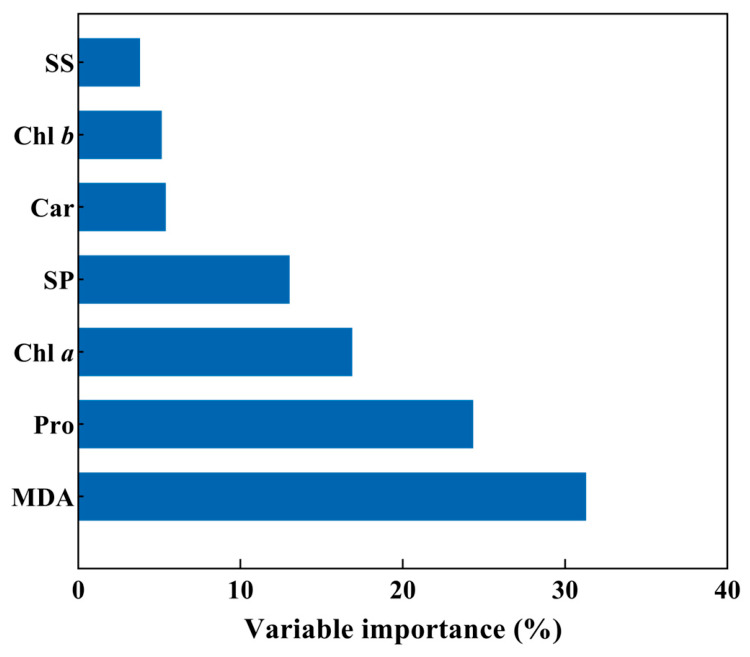
Relative importance of cell membrane permeability, osmoregulatory substances, and chloroplast pigments and cold tolerance to cold tolerance. Chl *a*, chlorophyll a; Chl *b*, chlorophyll b; Car, carotenoids; MDA, malondialdehyde; SS, soluble sugar; SP, soluble protein; Pro, proline.

A RF model was used to quantify the relative importance of cell membrane permeability, osmoregulatory substances, and chloroplast pigments in cold tolerance (Figure 5). The parameter settings for the random forest model are provided in Table S1, and the model evaluation results, which confirmed the model’s satisfactory accuracy, are presented in Table S2. The results of the random forest model showed that MDA, Pro, and Chl a explained the majority of the variation in REC across the seven holly species under different temperatures, accounting for 72.6%. Among these, MDA was identified as the most influential factor for REC, with a relative importance of 31.4%. In contrast, Car, Chl b, and SS exhibited relatively low importance in explaining REC, with their combined relative importance totaling only 14.38%.

## 4. Discussion

Evergreen *Ilex* species have high ornamental value, yet their distribution in cold regions is constrained by low temperatures. This study examines the interspecific differences in physiological responses of detached leaves from seven *Ilex* species under low-temperature stress and, based on these responses, provides a preliminary assessment of their cold tolerance, thereby providing a reference for the identification of cold-tolerant *Ilex* germplasm.

The cell membrane system constitutes the primary target of low-temperature damage in plants [25]. REC serves as a key indicator of membrane permeability changes, with lower temperatures inducing more severe cellular damage. In this study, *I. opaca* exhibited exceptional cold tolerance, with a REC value of 27.61% at −20 °C, which strongly aligns with Lyons’ membrane lipid phase transition hypothesis [26]. This hypothesis posits that an elevated proportion of unsaturated fatty acids (e.g., linoleic acid C18:2, linolenic acid C18:3) lowers the membrane lipid phase transition temperature, thereby preserving membrane fluidity under low-temperature stress. Similar patterns have been observed in *Arabidopsis*, where higher levels of unsaturated fatty acids are associated with enhanced cold tolerance [27].

As a primary product of membrane lipid peroxidation, MDA content reflects the extent of peroxidative damage [28]. In this study, a highly significant positive correlation was observed between REC and MDA content, suggesting that membrane lipid peroxidation may contribute to low-temperature injury. Previous studies on cold tolerance in olive (*Olea europaea* L.) reported that a cold-tolerant cultivar accumulated only 60% of the MDA content of cold-sensitive cultivars at −10 °C, a difference that was associated with a linolenic acid proportion of 52.3% in its membrane lipids [29]. In the present study, the minimal MDA accumulation observed in *I. opaca* at −20 °C might therefore be related to a relatively high proportion of unsaturated fatty acids in its membrane lipids. Direct biochemical evidence is still needed to support this hypothesis.

Osmotic regulation is an important physiological mechanism by which plants withstand low-temperature stress. Plants participate in the regulation of cellular osmotic balance through changes in the levels of osmotic regulators such as soluble sugars, soluble proteins, and proline, thereby resisting abiotic stress [30]. Previous studies have shown that under low-temperature stress, plants increase their levels of soluble sugars and soluble proteins, which help enhance cellular fluid concentration and water-holding capacity, thereby lowering the freezing point and contributing to membrane stability [16,17]. Consistent with these observations, the soluble sugar and soluble protein contents of the seven Ilex cultivars in the present study generally increased first and then decreased as temperature declined. Notably, in the cold-tolerant cultivars (*I. opaca, I. × cassine*, and *I.* ‘Nellie R. Stevens’), these levels were significantly higher and reached their maximum at lower temperatures compared to the other cultivars. Together, these results are consistent with the role of soluble sugars and soluble proteins in cold acclimation and membrane protection [30,31]. In addition, a significant negative correlation was observed between soluble sugar content and REC, suggesting that soluble sugars may play a dual role in cold tolerance: beyond osmotic adjustment, they may also contribute directly to membrane stabilization. A similar sugar–membrane interaction has been reported in Arctic willow (*Salix arctica*), where stem sucrose content reaches up to 40% of dry weight during winter [31]. Proline is an important osmotic regulator that helps maintain osmotic balance and stabilize cell structure under abiotic stress, and it also acts as a non-enzymatic antioxidant to remove reactive oxygen species [32]. In this study, low-temperature stress significantly increased proline levels in various Ilex cultivars, with a larger increase observed in cold-tolerant cultivars such as *I. × cassine* (a 98.16% increase at −20 °C). These observations suggest that higher proline levels may be associated with enhanced adaptability to low temperatures, potentially through reducing oxidative stress and increasing cellular osmotic potential [33]. Research on the cold resistance of grape (*Vitis vinifera* L.) rootstocks has similarly reported higher proline levels and high antioxidant enzyme activity under low-temperature stress [34]. It should be noted that leaf water content was not measured in this study, which should be considered when interpreting the osmotic data. Therefore, the observed changes in osmotic regulators may be partly influenced by passive concentration due to dehydration.

Chloroplast pigment responses to low-temperature stress varied substantially across *Ilex* species, reflecting interspecific differentiation in the stability of the photosynthetic apparatus. The continuous accumulation of pigments in *I. × cassine* from 5 °C to −15 °C (9.23% increase) may be related to adaptive changes in chloroplast structure and organization, as previously observed in cold-stressed *Arabidopsis* [35]. In contrast, the rapid degradation of pigments in *I. dabieshanens* may be associated with a reduced photoprotective capacity, which has been linked to increased susceptibility to photoinhibition under low-temperature stress [36]. Furthermore, a significant negative correlation was observed between MDA and Chl *a*, as well as between MDA and Chl *b*, while a significant positive correlation was observed between MDA and REC. These correlations suggest that membrane lipid peroxidation, photosynthetic pigment degradation, and membrane permeability disruption may occur together under low-temperature stress.

The apparent inconsistency among some physiological indicators—for example, the initial increase and subsequent decline of osmoregulatory substances versus the continuous rise in REC and MDA—reflects the asynchronous nature of plant responses to low-temperature stress. In the early stage of stress (from 5 °C to −5 °C or −10 °C), plants actively adjust osmotically by accumulating soluble sugars, soluble proteins, and proline during which membrane permeability remains relatively stable. As the temperature drops further (below −10 °C), membrane lipid peroxidation intensifies, leading to a sharp increase in REC and MDA, while the protective osmotic regulation systems gradually fail or become overwhelmed. Such asynchronous dynamics are common in stress physiology and highlight the need for a comprehensive multi-indicator evaluation approach to obtain an integrated cold tolerance ranking instead of relying on any single indicator.

Random forest analysis indicated that MDA, proline, and Chl *a* were closely associated with of cold tolerance, collectively explaining 72.6% of the variance in REC. Among these indicators, MDA exhibited the highest relative importance (31.4%), suggesting a potential link between membrane lipid peroxidation and freezing injury under acute cold stress. Previous studies have shown that LT_50_ predictions via logistic models generally support the conductivity method for relative cold tolerance comparisons among cultivars [12], with a lower LT_50_ typically indicating stronger cold tolerance [37]. However, in the present study, no inflection point was reached for I. opaca within the experimental temperature range (down to −20 °C). Consequently, its LT_50_ value could only be estimated by extrapolation and is of limited absolute accuracy, serving merely as a qualitative reference. Therefore, *I. opaca* was excluded from LT_50_-based comparisons. For the remaining six cultivars, the ranking based on LT_50_ showed some discrepancies when compared with other physiological indicators (e.g., the relative order of some intermediate cultivars differed). This suggests that LT_50_ may serve as a useful initial screening indicator, but a reliable assessment of cold tolerance ultimately requires the integration of multiple physiological parameters, including membrane integrity, osmoregulatory, and photosynthetic indicators.

This study has several limitations. Detached leaves cannot capture whole-plant cold acclimation or field overwintering responses, and therefore the results derived from this method does not directly translate to whole-plant field cold tolerance. Second, the rapid stepwise cooling (0.8 °C·min^−1^) and short-term exposure (2 h per temperature) simulate acute stress only, not natural winter cooling. Third, all measured parameters are indirect indicators (REC, MDA, osmoregulatory substances, and pigments) and do not directly reflect plant survival. Consequently, the results should be regarded as a preliminary physiological screening conducted under specific experimental conditions (detached leaves, acute stress). Given their correlational nature, these findings require field validation and do not directly support breeding or introduction recommendations without whole-plant field confirmation.

## 5. Conclusions

This study observed the physiological responses of detached leaves from seven evergreen *Ilex* species under acute low-temperature stress. Marked differences in stress responses were observed among the tested cultivars. Among these, *I. opaca* exhibited lower REC (27.61% at −20 °C) and lower MDA accumulation, along with relatively stable levels of osmotic regulators and photosynthetic pigments. Correlation and random forest analyses indicated that MDA, proline, and Chl *a* were associated with cold tolerance, collectively accounting for 72.6% of the variance in REC. The positive correlation between REC and MDA, together with the negative correlation between REC and proline, suggests underlying links between membrane lipid peroxidation, proline levels, and membrane stability under low-temperature stress. These observations reveal the physiological responses of evergreen *Ilex* species to acute low-temperature stress and provide a preliminary assessment of their cold tolerance.

## Figures and Tables

**Table 1:** Low-temperature semi-lethal temperature and logistic equations for seven *Ilex* species.

Breed	LT_50_	a	b	Logistic Equation	*R* ^2^
*I. opaca*	No inflection point reached; LT_50_ could not be calculated				
*I.* × *cassine*	−22.6	5.013	0.071	y = 100/(1 + 5.013e^−0.071x^)	0.833
*I.* × ‘China Girl’	−20.1	5.887	0.088	y = 100/(1 + 5.887e^−0.088x^)	0.822
*I.* ‘Nellie R.Stevens’	−19.8	5.464	0.086	y = 100/(1 + 5.464e^−0.086x^)	0.806
*I.* cornuta ‘Dwarf Burfordii’	−17.5	5.368	0.096	y = 100/(1 + 5.368e^−0.096x^)	0.810
*I. dabieshanensis*	−13.3	3.376	0.092	y = 100/(1 + 3.376e^−0.092x^)	0.826
*I.* cornuta Lindl. et Paxt. ‘Luteacarpus’	−11.9	4.764	0.131	y = 100/(1 + 4.764e^−0.131x^)	0.899

**Table 2:** Pearson correlation analysis of physiological indicators related to cold resistance in seven *Ilex* species.

Index	Chl *a*	Chl *b*	Car	MDA	Pro	SP	SS	REC
Chl *a*	1.000							
Chl *b*	0.528 **	1.000						
Car	0.139	−0.441 **	1.000					
MDA	−0.674 **	−0.163	−0.160	1.000				
Pro	0.260	0.340 *	−0.124	0.028	1.000			
SP	−0.154	−0.245	0.141	0.250	−0.156	1.000		
SS	−0.747 **	−0.648 **	0.224	0.505 **	0.001	0.245	1.000	
REC	−0.605 **	−0.351 *	−0.231	0.544 **	−0.415 **	0.494 **	0.233	1.000

Note: *: *p* < 0.05; **: *p* < 0.01. Chl *a*, chlorophyll a; Chl *b*, chlorophyll b; Car, carotenoids; MDA, malondialdehyde; SS, soluble sugar; SP, soluble protein; Pro, proline; REC, relative electrical conductivity.

## Data Availability

The original contributions presented in this study are included in the article/Supplementary Material. Further inquiries can be directed to the corresponding author.

## Supplementary Materials

The following supporting information can be downloaded at: https://www.mdpi.com/article/10.3390/plants15111751/s1. Table S1: Model parameter settings of random forest analysis; Table S2: Model evaluation results of random forest analysis.
