# Supplementary material for: Preliminary Cold Tolerance Evaluation of Seven Ilex Species Based on Physiological Responses of Detached Leaves to Acute Low-Temperature Stress
Source: Plants (Basel). 2026 Jun 4;15(11):1751. doi: 10.3390/plants15111751 (PMC13259453; doi:10.3390/plants15111751)
Supplement: Supplementary file 1 [file plants-15-01751-s001.zip › plants-4321015-supplementary.pdf]

Table S1. Model parameter settings of random forest analysis

Model	Parameter
Random Forest (RF)	n_estimator = np.range (0, 200, 10)<br>min_samples_leaf = 1<br>min_samples_split = 2<br>max_features = auto

Table S2. Model evaluation results of random forest analysis

Metric	Training set	Test set
MSE	25.4	227.57
RMSE	5.09	15.09
MAE	3.89	10.97
MAPE (%)	13.57	36.26
R^2	0.92	0.70
